# Technique of Administering Serum

**Published:** 1912-04

**Authors:** 


### Abstracts and Selections.

### Technique of Administering Serum.

1. *Lumbar Puncture.*—The first step in the administration of serum is "lumbar puncture," the passage of a needle into the spinal canal in the lumbar region. The needle employed for this purpose is preferably one made especially for the purpose, a fairly thick, stout, hollow needle, provided with an obturator to prevent stoppage and to strengthen the needle during its passage into the canal. A convenient needle, already sterilized, is furnished with each package of antimeningococcic serum as commercially supplied in this country.

As the procedure is not extremely painful, it is best performed, wherever possible, without general anesthesia. The question of employing an anesthetic must, however, be left largely to the discretion of the operator. In patients with marked opisthotonos, especially when restless or violently delirious, it is frequently better to employ a general anesthetic than to run the risk of failure or accident by attempting to proceed without one.

Rigid asepsis is absolutely essential to success. The same care should be used in cleansing the area of operation, the hands of the operators, instruments, etc., as would be employed in an aseptic laparotomy.

If the operation is done without anesthetic, there should be sufficient number of attendants to keep the patient quite still. If done under general anesthesia, there should be at least two persons besides the anesthetist.

The patient is laid upon the side with the back bowed. The puncture may be in the second, third, or fourth lumbar interspace. The site may be conveniently located as follows: Draw a line across the back, joining the highest points of the iliac crests. This line passes between the spinous processes of the fourth and fifth lumbar vertebræ, which, having been thus located, should be verified by counting. The puncture may be made midway between these processes, one-fourth to one-half inch from the mid line, the needle being directed forward, very slightly upward, and a little inward, so as to reach the dual sac just a little to the side of the median line, avoiding the veins located there.

If the needle is properly directed, its entry into the dual sac will be readily perceived by the sudden cessation of resistance. If it is improperly directed and impinges upon the bone, it should be withdrawn and redirected after a careful review of the landmarks. The distance to which the needle must be passed to reach the dual sac varies according to the age and musculature of the patient. In children it will be about two centimeters (three-fourths to one inch). In adults it may be as much as four to six centimeters (one and one-half to two and one-half inches).

When the needle is in place the obturator should be removed and the fluid which flows from the needle be received directly into a sterile flask or large test tube, so marked as to indicate at least approximately the amount (in cubic centimeters) of fluid withdrawn. It is important to know the amount of fluid withdrawn, as it has a bearing upon the amount of serum which may be safely injected. It is also important to have the fluid in a clean glass vessel where its gross appearance may be immediately noted, and in which it may be kept for bacteriological examination.

Ordinarily the fluid may be expected to flow freely in a stream under pressure. It should be withdrawn until the pressure is relieved and it flows only by drops. The amount withdrawn may vary considerably, from 10 to 150 c.c. or more. When possible a quantity equal at least to the amount of serum to be injected (30 to 60 c.c.) should be withdrawn; a larger amount if the pressure is high and the flow free. The fluid is characteristically turbid in cerebrospinal meningitis, sometimes purulent with flakes of fibrin.

Sometimes when a puncture seems to have been successful no fluid comes from the needle. In such cases the needle should be cautiously manipulated—the needle cleared thoroughly with the obturator, then pushed cautiously in a little farther or withdrawn slightly. After the fluid has started to flow it may cease because of the needle becoming clogged. The needle should, therefore, be thoroughly cleared with the obturator before the operator allows himself to be satisfied that the pressure of the fluid is sufficiently reduced. In some cases it may happen that no fluid at all is obtained, as when the case is more chronic and the meningeal exudate thick and fibrinous.

*Injection of the Serum.*—When a sufficient amount of fluid has been withdrawn the serum is injected through the same needle. The serum furnished by the American manufacturers is put up in syringes which may be connected aseptically with the puncture needle supplied. If the serum is not supplied in these syringes it should be drawn up from its receptacle into a carefully sterilized large syringe, previously tested with care to insure effective smooth working.

The serum should be injected very slowly, while the condition of the patient is carefully watched by an assistant. The amount to be injected depends upon several considerations. A child should receive ordinarily about 15 c.c. to 30 c.c., an adult from 30 c.c. to 60 c.c., at the first injection. These amounts may ordinarily be introduced with little fear or difficulty if the amount of fluid withdrawn is equal to or greater than this. Quite often, however, the amount of fluid obtainable is less than the amount of serum which it is desirable to administer. In such cases one must carefully note the resistance offered to the injection, must avoid undue pressure, and must watch closely for untoward symptoms, but in their absence need not be deterred from administering the full dose.

*Course of Treatment.*—Antimeningococcic serum has not as yet been standardized with an accuracy comparable to that used in the standardization of antidiphtheritic serum. Greater variations are therefore to be expected in the potency of different samples. Also its action differs essentially from that of the purely antitoxic sera; hence in its administration one must be guided by somewhat different considerations, must be guided to a greater extent by results rather than by dosage.

Experience has shown that the best results can be obtained only by repeated injections. Although surprising results are often obtained from a single injection, unless the destruction of the meningococci is complete relapse is likely to recur. It is, therefore, recommended by Netter and Debré as a routine procedure to give at least three or four injections of full doses of serum at intervals of twenty-four hours, and after that to be guided by the clinical signs and changes in the cerebrospinal fluid. Ordinarily after three or four daily injections the fever will have fallen, the rigidity be relaxed, and the mental condition of the patient markedly improved. Since, however, these signs do not necessarily indicate complete recovery, it is highly important to make observations at each injection upon the cerebrospinal fluid and to be guided largely by its appearance. Under the influence of the serum the cerebrospinal fluid becomes clearer, the large adventitial cells diminish, the leucocytes are relatively increased and less degenerated, lymphocytes to some extent replace the polymorpho-nuclear leucocytes, the number of meningococci present in the fluid is diminished, and those that remain are found to be degenerated. Under favorably progressing treatment the meningococci disappear completely after from one to four injections. Levy gives the following figures as to the disappearance of meningococci in the cerebrospinal fluid. Meningococci could no longer be demonstrated:

"In 18 cases after a single injection; in 33 cases after 2 injections; in 35 cases after 3 injections; in 14 cases after 4 injections; in 9 cases after 5 injections; in 4 cases after 6 injections; in 1 case after 11 injections."

While these results illustrate the remarkable action of the serum in destroying the meningococci, they also show the necessity of repeating the injections as recommended and of being guided by the examination of the cerebrospinal fluid in continuing injections after the third or fourth.

Too much emphasis can not be laid upon the point that the treatment of cerebrospinal meningitis must be vigorous, with full doses, continued until a cure is effected. A large part of the failures to get satisfactory results have been from ignorance or neglect of this important point, from the tendency to let well enough alone, and to wait for the full effect of each dose before following it up with another.

It may happen that the inflammatory process may be largely localized in the ventricles of the brain, and the communication between these and the spinal subarachnoid space obliterated. In such cases severe symptoms will continue even after the cerebrospinal fluid appears free from meningococci, and intraspinal injections will be ineffective. It is advisable in these cases to make the injections directly into the ventricles of the brain, bilaterally if necessary. The indications for this procedure are prolonged subacute or chronic course of the disease, with the signs of acute hydrocephalus, viz., intensified stupor, headache, cerebral vomiting, slowing and irregularity of the pulse, "choked disc," and the failure of intraspinal injections to relieve the conditions. The procedure itself is a surgical operation, which can not be described here. —*U. S. P. H. Reports.*

